# New records of Lumbricidae and Collembola in anthropogenic soils of East European tundra

**DOI:** 10.3897/zookeys.885.37279

**Published:** 2019-11-04

**Authors:** Alla A. Kolesnikova, Maria A. Baturina, Dmitry M. Shadrin, Tatyana N. Konakova, Anastasia A. Taskaeva

**Affiliations:** 1 Institute of Biology of Komi Scientific Centre of the Ural Branch of the Russian Academy of Sciences, Kommunisticheskaja, 28, RU-167000, Syktyvkar, Russia Institute of Biology of Komi Scientific Centre of the Ural Branch of the Russian Academy of Sciences Syktyvkar Russia

**Keywords:** DNA barcoding, earthworms, springtails

## Abstract

The terrestrial environment of the East European tundra consists of a mosaic of habitat types. In addition to the natural habitat diversity, various human-influenced types may occur. In the town of Vorkuta, Komi Republic, Russia the manure-enriched soils near hydrogen sulfide springs were observed. This site represents an unusually nutrient-rich location with considerable development of organic soils, in contrast to the naturally forming soils in East European tundra which are typically thin and nutrient poor. In these organic soils, two species of Lumbricidae and two species of Collembola previously not recorded from the natural ecosystems in the study area of research territory were found. One earthworm species, *Dendrodrilus rubidus tenuis*, is likely to have been introduced. The presence of the three other species (*Eiseniella tetraedra*, *Folsomia fimetaria*, and *Proisotoma minuta*) is quite natural in East European tundra and such anthropogenic soils with high organic content may be a good habitat for them.

## Introduction

Tundra ecosystems are characterized by a low number of species, together with low productivity (Olejniczak et al. 2018; Phillips et al. 2019). Unfortunately, these ecosystems are known to be susceptible to disturbances, particularly if they are of anthropogenic origin (Coulson et al. 2015; Mikola et al. 2014). Extraction of natural resources, air pollution, human settlements, and tourism all affect tundra soils, vegetation, and thus soil organisms (Chapin et al. 2015; Coulson et al. 2013a, 2015; Olejniczak et al. 2018). Human activity is usually accompanied by a reduction in biodiversity, but it can also contribute to increased biodiversity of polar ecosystems by the introduction of non-indigenous species (Coulson et al. 2015). Man-made habitats are often more suitable and successful for colonists than natural habitats (Ødegaard and Tømmerås 2000). More than 65 % (1040 species) of alien arthropod species in Europe are associated with human-made habitats, especially parks and gardens, human settlements, and agricultural lands (Lopez-Vaamonde et al. 2010). Habitats of this type include accumulations of dead plant material and manure heaps of animal origin. These rich organic layers provide climatically stable living spaces and food for different groups of animals. One such habitat is associated with the anthropogenic soils in the Russian mining town of Vorkuta in the East European tundra.

The East European tundra is the territory bound by the Kanin Peninsula on the west and the Kara River Basin in the east between 67–71°N and 50–65°E. This territory is diverse, ranging from polar deserts with extremely low plant cover to moss, dwarf birch, willow, and forest tundra. The heterogeneity is also reflected in the soil animal fauna, where often clear relationships can be observed between vegetation cover and invertebrate species diversity (Coulson et al. 2013b).

The earthworm fauna in this territory is very poor and includes only three species: *Dendrobaena octaedra* (Savigny, 1826), *Lumbricus rubellus* Hoffmeister, 1843, and *Eisenia nordenskioldi nordenskioldi* (Eisen, 1879), which widely distributed in the study area because of their ability to withstand soil freezing (Makarova and Kolesnikova 2019; Shekhovtsov et al. 2018). This fauna can be seen as a marginal element compared to the more diverse earthworm fauna of Fennoscandia, where eight species are found to the north of the 65^th^ parallel north (Terhivuo 1988) and Kola Peninsula, where six species were found beyond the Arctic Circle (Zenkova and Rapoport 2011; Rybalov and Kamaev 2012). Both these territories are heated by the Gulf Stream and were covered by glacial sheets that erased much of the fauna (Hewitt 2000). Therefore, endemic species are lacking here and the species present are post-glacial immigrants, that have invaded either spontaneously or in association with human activities. East European tundra had only limited glaciation (the furthest glacial maximum and last glacial maximum of Quaternary glaciation) but a harsher climate, and some populations of earthworms could survive or only recently colonize this territory (Shekhovtsov et al. 2018). However, the macro-scale distribution of earthworm species shows little connection to the pattern of the last glaciation. The earthworm fauna of the northern Russian plain is composed mainly of peregrine species of European origin (Tiunov et al. 2006).

On the other hand, springtails play an important role in tundra ecosystems as they affect the processes of humification and mineralization of organic matter (Babenko 2012; Coulson et al. 2013a, b, 2015; Olejniczak et al. 2018). A total of 192 collembolan species is registered for the territory of the East European tundra. Among them, 30 species are unique and absent from neighboring regions, due to a number of ecological factors (Babenko et al. 2017). However, there is no information about the invertebrate fauna, native or introduced in anthropogenic soils.

The aim of this study was to assess the distribution of earthworms and springtails that are new in the Eastern European tundra, and test the working hypothesis that these records of these species are confined to anthropogenic soils near hydrogen sulfide sources beyond the Arctic Circle.

## Materials and methods

**Figure 1.**
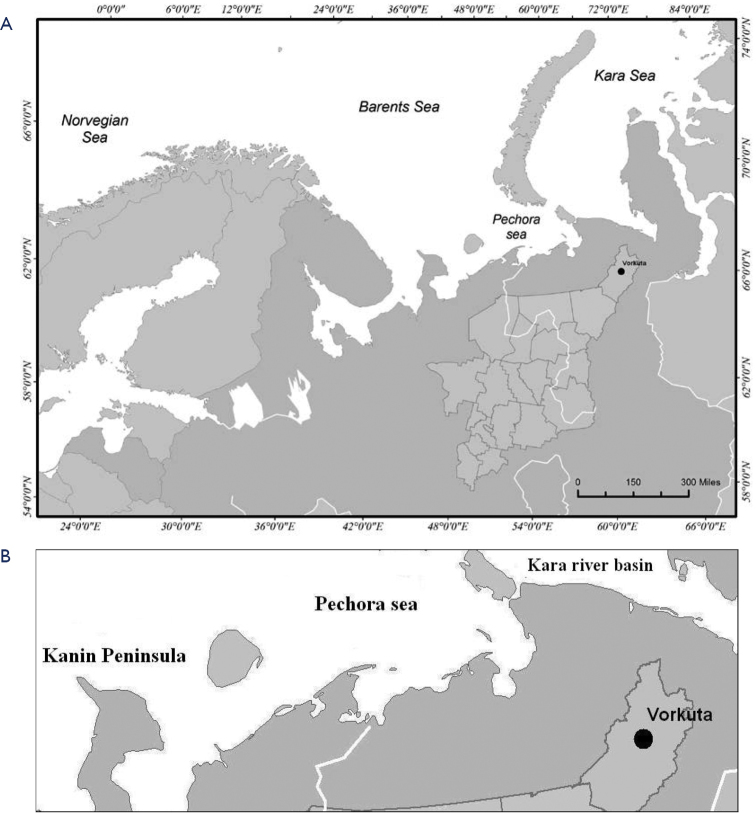
The location of Komi Republic, Russia (**A**) and East European tundra (**B**).

Soil samples were collected from the sides of a gully formed in the organic soils accumulated near hydrogen sulfide brooks (67°29'N, 64°02'E) in Vorkuta in Komi Republic, Russia (Fig. 1). The soil formed layers 10 cm thick which were created from a mixture of discarded poultry factory food stores, city hospital, and railway depot (Getsen 2011). Due to the warm municipal sewage that is discharged into the stream, in winter it does not freeze. Analysis of physical and chemical properties showed that the soils are characterized by a neutral pH (pH = 7.3) with contents of nitrogen (N_tot_ = 1.3 %), carbon (C_tot_ = 21 %) and narrow C:N ratio. A similar picture was obtained for postagrogenic soils in the European Northeast of Russia. On the contrary, an acid reaction, high content of carbon (C_tot_ = 32 %), low content of nitrogen (N_tot_ < 1 %) and wide C:N ratio were recorded in tundra soils, which indicates a low enrichment of soil organic matter with nitrogen and a weak degree of decomposition (Taskaeva et al. 2019).

Twenty soil samples 10 × 10 × 10 cm were taken near hydrogen sulfide springs in Vorkuta on July 2017 and August 2018. The soil samples were immediately returned to the Institute of Biology, Syktyvkar, Komi Republic, and placed in Tullgren soil extractors within 24 h of sampling. The soil fauna was extracted under 40 W light bulbs into 96 % alcohol for seven days until the soil was completely dry. Accounting of earthworms near hydrogen sulfide springs by manual sorting of soil samples 25 × 25 × 10 cm was not carried out because of the small area of the studied plots. Moreover, the recent results showed that the earthworm abundance estimated by the Tullgren funnel extraction method exceeds the values obtained through manual sorting of the samples by an order of magnitude (Makarova and Kolesnikova 2019). The Collembola were identified to species by morphological characters (Fjellberg 2007; Potapov 2001). Identification of Lumbricidae was performed according to morphological characters provided by Vsevolodova-Perel (1997) and Timm (2009), as well as DNA barcoding for immature individuals.

DNA was extracted from several caudal segments using 6 % Chelex 100 DNA extraction kits (Sigma-Aldrich, USA). A fragment of the COI gene was amplified in 50 μl of mixture containing 10 μl ScreenMix (Eurogen, Russia), 10 μl of each primer (0.3 μM) (Eurogen, Russia), 18 μl ddH2O (Ambion, USA), and 2 μl DNA template (1÷100 ng). Two universal primers designed for invertebrate organisms were used to amplify the site of the COI fragment: LCO1490m (5’-TACTC-AACAA-ATCAC-AAAGA-TATTG-G-3’; modified from Folmer et al. 1994) and HCO2198m (5’-TAAAC-TTCAG-GGTGA-CCAAA-AAATC-A-3’; Folmer et al. 1994). Sequencing was performed using the equipment of The Center for Collective Use “Molecular Biology” of the Institute of Biology of the Komi Scientific Center of the Ural Branch of the Russian Academy of Sciences. The GenBank database was used for sequence identification. Sequence alignment (automatic and manual) and choice of an evolutionary model were performed using the MEGA 7.0 program (Kumar et al. 2016). We applied Neighbor Joining (NJ) and Maximum Likelihood (ML) estimation methods. Since both methods resulted in a similar outcome, here we present the trees obtained by the NJ method only. The default parameters for tree building were selected. The Tamura-Nei model was selected for ML analysis. To align sequences, we used the ClustalW algorithm and the robustness of the resulting lineages was tested by bootstrap analysis with 1,000 replications. For NJ we used the p-distance model. Sequences were sent to GenBank (accession numbers and BINs are presented in Table 1).

**Table 1.** Invertebrate species previously unrecorded in East European tundra collected from the anthropogenic soils near a hydrogen sulfide spring in Vorkuta.

Class	Species	Records in East European tundra	Records beyond Arctic Circle in Europe	Distribution	GenBank accession number
Oligochaeta	*Dendrodrilus rubidus tenuis* (Eisen 1874)	Vorkuta (67°29'N, 64°02'E)	Iceland? (Blakemore 2007); Svalbard (Coulson et al. 2013a, b, form is unknown); Fennoscandia (Terhivuo 1988, form is unknown); Kola Peninsula (Zenkova and Rapoport 2011)	Holarctic. Records from the southern hemisphere	MH410150
	*Eiseniella tetraedra* (Savigny, 1826)	Pechora delta (68°11'N, 53°82'E) Pymvashor (67°09'N, 60°51'E) Kharbey lakes (67°58'N, 62°34'E) Vorkuta (67°29'N, 64°02'E)	Iceland (Blakemore 2007); Fennoscandia (Terhivuo 1988); Kola Peninsula (Timm and Abarenkov 2018)	Cosmopolitan	MH410149
Collembola	*Folsomia fimetaria* (Linnaeus, 1758)	Vorkuta (67°29'N, 64°02'E)	Greenland (Babenko and Fjellberg 2006) Svalbard (Coulson et al. 2013a, b) Fennoscandia (Fjellberg 2007) Kola Peninsula (Babenko 2012)	Holarctic	–
	*Proisotoma minuta* (Tullberg, 1871)	Vorkuta (67°29'N, 64°02'E)	Fennoscandia (Fjellberg 2007)	Cosmopolitan	–

## Results and discussion

Two species of lumbricids, *Dendrodrilus rubidus tenuis* (Eisen, 1874) and *Eiseniella tetraedra* (Savigny, 1826), and two species of springtails, *Folsomia fimetaria* (Linnaeus, 1758) and *Proisotoma minuta* (Tullberg, 1871), not previously recorded from East European tundra were collected. Their records on this territory and beyond the Arctic Circle are shown at Table 1.

**Figure 2.**
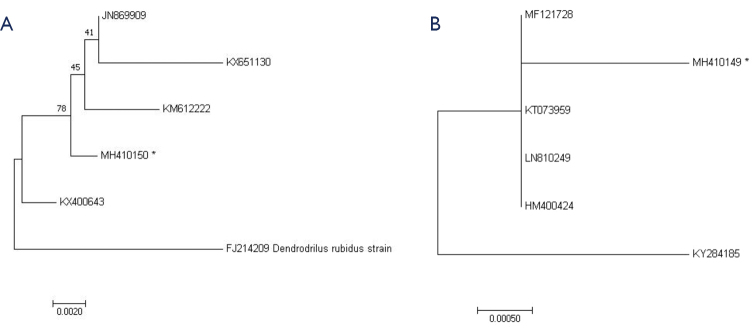
Phylogenetic tree constructed by the NJ method on the basis of a comparison of the nucleotide sequences of the COI gene of *Dendrodrilus rubidus tenuis* (**A**) and *Eiseniella tetraedra* (**B**) species from other regions (Austria JN869909, KT073959; USA KX651130; Canada KM612222, HM400424; Russia, Southern Kuriles KX400643; Finland FJ214209; France MF121728; Switzerland LN810249; Spain KY284185). * the numbers of our data sequences from Komi Republic.

Both species of Lumbricidae are widespread, including records from the southern hemisphere. *Dendrodrilus rubidus* is found on every continent except Antarctica and inhabits not only continents, but also many islands. It is often found in wet and moist soils by rivers, brooks, and springs, and thrives in compost heaps and in a variety of man-made habitats including rich soils close to settlements (Terhivuo 1988). It was recently found at 70°N (eastern Finmark, Norway) and appears to be common throughout the northern mainland of Norway and in Svalbard cowsheds (Coulson et al. 2013a, 2013b). In our study we found the subspecies *Dendrodrilus rubidus tenuis*. COI sequence of one individual was identified and differed from a sequence of this species from Canada (KM612222) by two substitutions and from Russia, Southern Kuriles (KX400643) by four substitutions (Fig. 2A). This species is abundant in temperate ecosystems of Eastern Europe, cultivated areas in the taiga zone of European North-East, southern part of Siberia, stone birch-forests of Kamchatka (Vsevolodova-Perel 1988, 1997; Shekhovtsov et al. 2014; Akulova et al. 2017) and in greenhouses in northern settlements of Yakutia, Magadan oblast, and Chukotka (Berman et al. 2010). In winter, for example, greenhouses are not heated and the temperature may descend to below -40 °C. Nonetheless, the cocoons are viable at temperatures lower than – 40 °C (Berman et al. 2010), but adults cannot survive exposure below -4 °C (Meshcheryakova and Bulakhova 2014). Considering the harsh winter conditions in Vorkuta, it will probably be restricted to areas with local enrichments of organic matter to provide protection against low air temperatures, such as anthropogenic soils. In our study it was found only in built-up areas, where the soil does not freeze in winter due to the warming action of some shelters (warm municipal wastes, poultry factory storages, etc.). Previously, beyond the Arctic Circle, *D. rubidus tenuis* was registered only in Khibiny Mountains (Kola Peninsula), where it was found in soils with a pH > 5 and high organic matter content (Zenkova and Rapoport 2011).

*Eiseniella tetraedra* is a cosmopolitan earthworm widely distributed in the Old and New World countries (Terhivuo et al. 2011). COI sequences of two *E. tetraedra* individuals were identified and differed from sequences of this species by only one substitution (Fig. 2B) from the most closely related GenBank entries, i.e. from France (MF121728), Austria (KT073959), Switzerland (LN810249), and Canada (HM400424). It is abundant in wet and moist alluvial shore soils, fucoid wrack beds, the banks of rivers and brooks, and it can be found in waterlogged or even limnic habitats such as the bottoms of rivers, brooks, and springs (Terhivuo 1988). Among all species of lumbricids it is the most resistant to flooding (Plum 2005). It is not directly linked with human culture and is not intentionally transported by human activity (Terhivuo et al. 2011), but it is a “key” species in anthropogenic habitats, the activity of which determines the character of soil dynamics (Barne and Striganova 2005). Considering its widespread distribution and parthenogenic reproduction, allowing it to settle and colonize quickly, *E. tetraedra* is an interesting object for studying biogeography and genetic diversity in soil, aquatic, and ecotone systems (de Sosa et al. 2017). In natural habitats, these worms live in large colonies (Barne and Striganova 2005) and can reach up to 1000 ind./m^2^ (Malevich 1959). Despite insignificant resistance to cold, its dispersal is determined by its amphibiotic nature: it can successfully overwinter only in talik or waterlogged soils, which only freeze in winter (Meshcheryakova and Berman 2014). It was previously found in Pechora River delta (Baturina 2018) and other aquatic ecosystems in East European tundra (Table 1).

Two species of Collembola previously unrecorded in East European tundra were found, *Folsomia fimetaria* and *Proisotoma minuta*. Both of these are typical members of a fauna associated with soils having a high organic content such as compost, garden soil, fucoid wrack beds along seashores, and stream banks. They are very seldom found in temperate forests in the European part of Russia. Due to their ecological preferences, they could be artificially introduced (Potapov 2001). For example, in Iceland and Fennoscandia *F. fimetaria* is often found in organic soils along seashores (Fjellberg 2007); in Kola Peninsula it was found in anthropogenic stations heavily enriched with organic matter (Babenko 2012). This species was recently found in cowsheds soils in Svalbard (Coulson et al. 2013a). It is a good test subject for chemical studies (Holmstrup and Krogh 1996). *Proisotoma minuta* is also very tolerant of unfavorable conditions; it prefers roots infected by several phytopathogenic fungi, but the preference does depend on the species of fungi (Potapov 2001). The preliminary list of Collembola is presented in Appendix 1.

Three of the four new species records for East European tundra observed here appear to be not currently invasive. However, our molecular genetic analysis of the earthworm *D. rubidus tenuis* that was found in Vorkuta suggests that our sample of this species potentially invasive in nutrient-high habitats. In contrast to the natural soils, the anthropogenic soils provide a nutrient-rich, organic soil (contents of N = 1.3 % and C = 21 %) with excellent water-holding properties and with a cool moist environment during the summer but beneficially altered via human activities. The brook Vodny, in the area of which the studies were conducted, does not freeze as it depends on warm municipal and domestic waste waters flowing into it. Thus, soils with high organic content can stay warm throughout the season as a result of a continuous fermentation process. Consequently, species preferring warm soils probably do not suffer from chilling injuries (Bale 1993) during the winter in Vorkuta, despite the fact that their temperature tolerances are adapted to a warmer climate (Ødegaard and Tømmerås 2000). However, both species of lumbricids *D. rubidus tenuis* and *E. tetraedra* are cold tolerant only in the cocoon stage, while adult worms die at − 1 °C to − 3 °C (Berman et al. 2010; Meshcheryakova and Berman 2014). This means that they have adapted to complete their life cycle in the short warm period. Both species of springtails *F. fimetaria* and *P. minuta* cannot be called aliens, because their presence was also noted in anthropogenic soils in tundra of other regions. To conclude, it is evident that despite not tolerating the cold, all four newly recorded species are still capable of living in anthropogenic environments in cold climates.

## Appendix 1

**Table:** Preliminary list of Collembola.

Order	Family	Species
Poduromorpha	Onychiuridae	*Protaphorura* sp.
	Hypogastruridae	*Ceratophysella denticulata* (Bagnall, 1941)
	Neanuridae	*Friesea truncata* Cassagnau, 1958
		*Pseudachorutes subcrassus* Tullberg, 1871
Entomobryomorpha	Isotomidae	*Desoria blufusata* (Fjellberg, 1978)
		*Desoria breviseta* Potapov, 2017
		*Desoria* sp.
		*Folsomia amplissima* Potapov & Babenko, 2000
		*Folsomia fimetaria* (Linnaeus, 1758)
		*Folsomia* sp.
		*Isotoma anglicana* Lubbock, 1873
		*Isotoma viridis* Bourlet, 1839
		*Isotomiella minor* (Schäffer, 1896)
		*Parisotoma notabilis* (Schäffer, 1896)
		*Proisotoma minuta* (Tullberg, 1871)
Symphypleona	Sminthurididae	*Sminthurides aquaticus* (Bourlet, 1842)
		*Sphaeridia pumilis* (Krausbauer, 1898)
	Katiannidae	*Sminthurinus aureus* (Lubbock, 1862)
